# Crystal structures of 2-meth­oxy­isoindoline-1,3-dione, 1,3-dioxoisoindolin-2-yl methyl carbonate and 1,3-dioxo-2,3-di­hydro-1*H*-benzo[*de*]isoquinolin-2-yl methyl carbonate: three anti­convulsant compounds

**DOI:** 10.1107/S1600536814023769

**Published:** 2014-11-05

**Authors:** Fortune Ezemobi, Henry North, Kenneth R. Scott, Anthohy K. Wutoh, Ray J. Butcher

**Affiliations:** a Department of Biology, College of Arts & Sciences, Howard University, 415 College Street NW, Washington, DC 20059, USA; b Department of Pharmaceutical Sciences, College of Pharmacy, Harding University, 915 E. Market Avenue, Box 12330, Searcy, Arkansas 72149, USA; c Department of Pharmaceutical Sciences, College of Pharmacy, Howard University, 2300 4th Street, NW, Washington, DC 20059, USA; d Department of Chemistry, Howard University, 525 College Street NW, Washington, DC 20059, USA

**Keywords:** crystal structure, anti­convulsant, isoindoline, iso­quinoline, indoline

## Abstract

The title compounds, C_9_H_7_NO_3_, (1), C_10_H_7_NO_5_, (2), and C_14_H_9_NO_5_, (3), are three potentially anti­convulsant compounds. Compounds (1) and (2) are isoindoline derivatives and (3) is an iso­quinoline derivative. Compounds (2) and (3) crystallize with two independent mol­ecules (*A* and *B*) in their asymmetric units. In all three cases, the isoindoline and benzoiso­quinoline moieties are planar [r.m.s. deviations are 0.021 Å for (1), 0.04 and 0.018 Å for (2), and 0.033 and 0.041 Å for (3)]. The substituents attached to the N atom are almost perpendicular to the mean planes of the heterocycles, with dihedral angles of 89.7 (3)° for the N—O—C_meth­yl_ group in (1), 71.01 (4) and 80.00 (4)° for the N—O—C(=O)O—C_meth­yl_ groups in (2), and 75.62 (14) and 74.13 (4)° for the same groups in (3). In the crystal of (1), there are unusual inter­molecular C=O⋯C contacts of 2.794 (1) and 2.873 (1) Å present in mol­ecules *A* and *B*, respectively. There are also C—H⋯O hydrogen bonds and π–π inter­actions [inter-centroid distance = 3.407 (3) Å] present, forming slabs lying parallel to (001). In the crystal of (2), the *A* and *B* mol­ecules are linked by C—H⋯O hydrogen bonds, forming slabs parallel to (10-1), which are in turn linked *via* a number of π–π inter­actions [the most significant centroid–centroid distances are 3.4202 (7) and 3.5445 (7) Å], forming a three-dimensional structure. In the crystal of (3), the *A* and *B* mol­ecules are linked *via* C—H⋯O hydrogen bonds, forming a three-dimensional structure, which is consolidated by π–π inter­actions [the most significant inter-centroid distances are 3.575 (3) and 3.578 (3) Å].

## Abstract 2

In three potentially anti­convulsant compounds, of which two are isoindoline derivatives and one an iso­quinoline derivative, the central moiety is planar. In the crystals of all three compounds, there are C—H⋯O hydrogen bonds present linking the mol­ecules into two-dimensional slabs for the isoindoline derivatives, and into a three-dimensional framework for the iso­quinoline derivative.

## Chemical context   

Traumatic brain injury (TBI) is a neurological disorder that is defined as damage to the brain resulting from external mechanical force, including accelerating, decelerating and rotating forces (Langlois *et al.*, 2003 ▶, 2005 ▶; Ashman *et al.*, 2006 ▶; Coronado *et al.*, 2011 ▶). TBI also exacerbates seizure severity in individuals with pre-existing epilepsy (Ferraro *et al.*, 1999 ▶), being one example of the process of epileptogenesis (Christensen *et al.*, 2009 ▶). In this context, it has been demonstrated that early lesions in the central nervous system (CNS) alter the transport dynamic of the blood–brain barrier (BBB) and deteriorate the balance of the inhibitory and excitatory neurotransmitter system (Scantlebury *et al.*, 2005 ▶]. This neuronal dysfunction predisposes to subsequent development of spontaneous recurrent seizures in the presence of prior subtle brain malformation (Love, 2005 ▶].
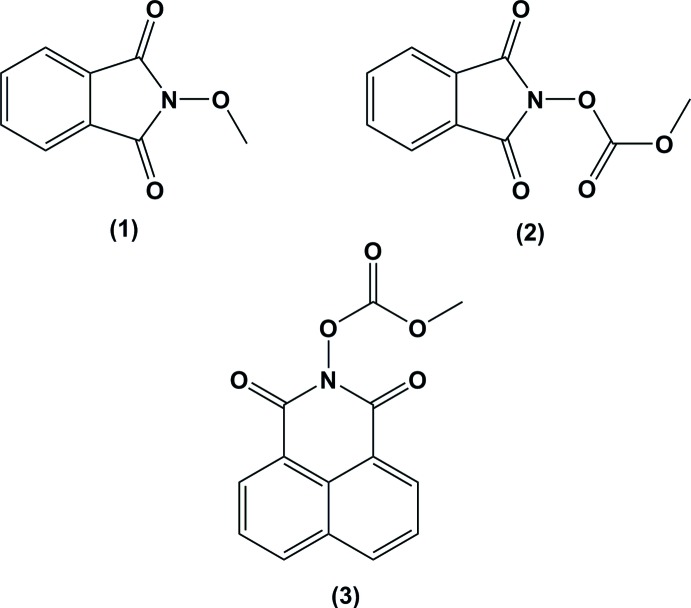


TBI is the major cause of death in young individuals (14–24 years) from industrialized countries, with head injuries accounting for 25–33% of all trauma-related deaths (Abdul-Muneer *et al.*, 2014 ▶). Disorders like memory loss, depression and seizures are some of the side effects to TBI. TBI affects people over 75 years of age because of falls and of 17–25 years of age because of accidents (Langlois *et al.*, 2003 ▶, 2005 ▶; Ashman *et al.*, 2006 ▶; Coronado *et al.*, 2011 ▶). At present, there are no effective treatments available for TBI and there is thus a critical need to develop novel and effective strategies to alter the disease course. As indicated above, this health condition is quite similar to epilepsy in some instances and thus our earlier work (Alexander *et al.*, 2013 ▶; Jackson *et al.*, 2012 ▶; Edafiogho *et al.*, 2007 ▶) on developing anti­convulsant compounds for the treatment of epilepsy is relevant.

Our research on pharmacologically active compounds is a multi-pronged approach, which involves synthesis, chemical characterization, computer modeling, pharmacological evaluation, and structure determination (North *et al.*, 2012 ▶; Gibson *et al.*, 2009 ▶). From this comprehensive approach, structure–activity correlations can be made to improve the existing pharmacologically active compounds. From our studies, we identified three imido­oxy derivatives as potential drug candidates for TBI that underwent anti­convulsant evaluation to test their ability to inhibit the onset of seizures in the *in vivo* MES, scPTZ test models. The MES (maximal electroshock seizure evaluation) test presented activity in animals in phase 1 testing.

2-Meth­oxy­isoindoline-1,3-dione, (1), studied by X-ray techniques, was inactive in MES and scPTZ in mice, but showed MES protection in rat studies at 50 mg kg^−1^ at 4 h and also protected 1/4 mice at three different time inter­vals (0.50, 1 and 2 h) in the 6 Hz test (Jackson, 2009 ▶). For scPTZ studies, the compound was Class III (no activity at 300 mg kg^−1^). The compound is a dual MES/6Hz active compound. Compounds (2) and (3) showed similar activity.

**Figure 1:**
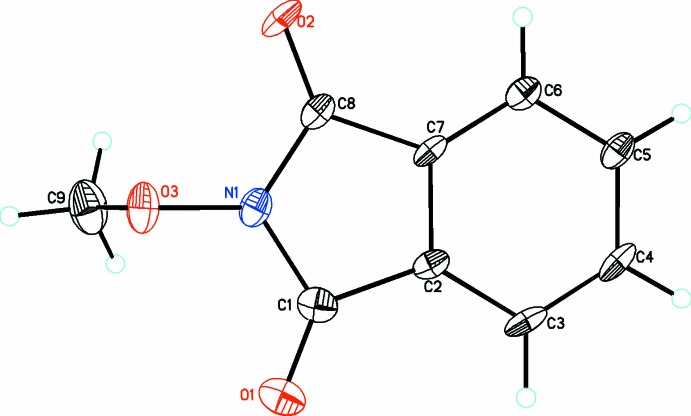
The mol­ecular structure of compound (1), with atom labelling. Displacement ellipsoids are drawn at the 30% probability level.

**Figure 2:**
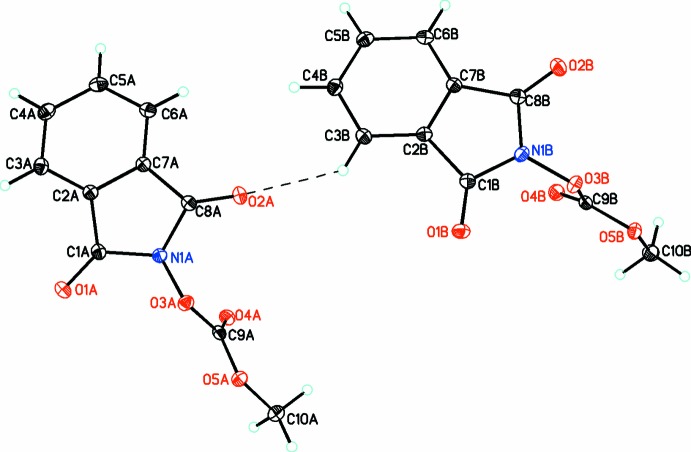
The mol­ecular structures of the two independent mol­ecules (*A* and *B*) of compound (2), with atom labelling. Displacement ellipsoids are drawn at the 30% probability level. The C—H⋯O hydrogen bond is shown as a dashed line (see Table 2 ▶ for details).

**Figure 3:**
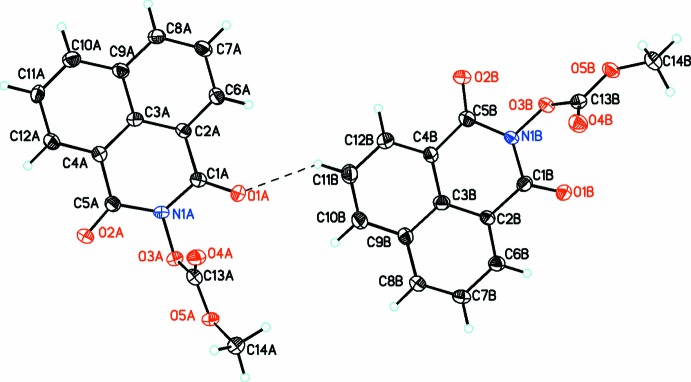
The mol­ecular structures of the two independent mol­ecules (*A* and *B*) of compound (3), with atom labelling. Displacement ellipsoids are drawn at the 30% probability level. The C—H⋯O hydrogen bond is shown as a dashed line (see Table 3 ▶ for details).

The title compounds, containing either an isoindoline-1,3-dione moiety, (1) (Fig. 1 ▶) and (2) (Fig. 2 ▶), or an iso­quinoline-1,3-dione moiety, (3) (Fig. 3 ▶), have been studied extensively for their anti­convulsant effects with promising results. Herein, we report on the crystal structures of these new structurally related compounds.

## Structural commentary   

In compound (1), the isoindoline ring is planar [r.m.s. deviation = 0.017 (4) Å]. The meth­oxy O atom, O3, deviates from this plane by 0.176 (6) Å while the methyl C atom, C9, is out of the plane by 1.105 (9) Å. The meth­oxy substituent is oriented almost perpendicular to the indoline ring with the dihedral angle between the mean planes of the indoline ring and the meth­oxy substituent being 89.7 (3)°.

In compound (2), there are two mol­ecules (*A* and *B*) in the asymmetric unit. The isoindoline ring is planar [r.m.s. deviation = 0.0327 (9) for *A* and 0.0147 (9) Å for *B*] with the dione O atoms significantly out of the plane for mol­ecule *A* but not for mol­ecule *B* [0.172 (1) and 0.123 (1) Å for atoms O1 and O2, respectively, in *A* but by only 0.013 (1) and 0.002 (1) Å, respectively, in *B*]. The carbonato moiety is planar in both mol­ecules [r.m.s. deviations of 0.0066 (2) and 0.0027 (5) Å for *A* and *B*, respectively] and makes dihedral angles of 71.50 (3) and 80.03 (4)° with the benzoiso­quinoline ring in *A* and *B*, respectively, indicating that these substituents are oriented almost perpendicular to the benzoiso­quinoline ring system.

In compound (3), there are also two mol­ecules (*A* and *B*) in the asymmetric unit. In both mol­ecules, the benzoiso­quinoline ring systems are planar (r.m.s. deviations for *A* and *B* = 0.033 and 0.015 Å, respectively). The meth­oxy O atom deviates from this plane by 0.126 (1) for atom O5*A* in *A* and 0.156 (1) Å for atom O5*B* in *B*. The methyl carbonate moieties are planar [r.m.s. deviations of 0.007 (1) and 0.003 (1) Å for *A* and *B*, respectively] and these substituents are oriented almost perpendicular to the iso­quinoline rings, making dihedral angles of 71.50 (3) and 80.04 (4)° for *A* and *B*, respectively. As in (2), these dihedral angles are significantly smaller than that found for (1).

## Supra­molecular features   

**Figure 4:**
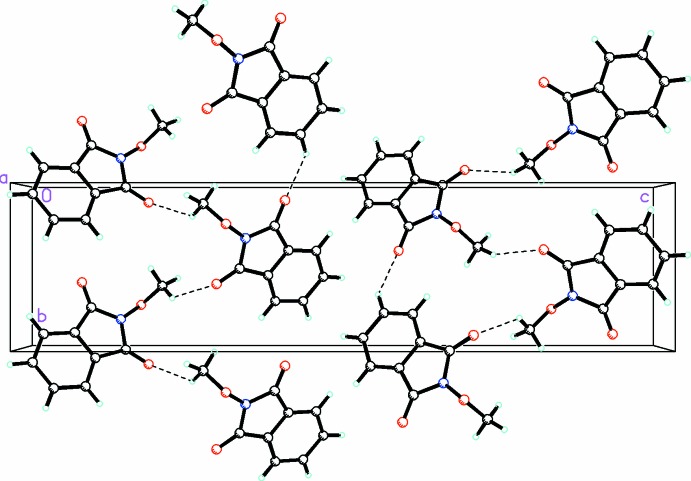
A view along the *a* axis of the crystal packing of compound (1), showing the formation of the three-dimensional array by an extensive network of C—H⋯O hydrogen bonds (shown as dashed lines; see Table 1 ▶ for details).

In the crystal of (1), there are C—H⋯O hydrogen bonds (Fig. 4 ▶ and Table 1 ▶) and π–π inter­actions present, forming slabs lying parallel to (001) [*Cg*1⋯*Cg*2^i,ii^ = 3.407 (3) Å; *Cg*1 and *Cg*2 are the centroids of rings N1/C1/C2/C7/C8 and C2–C7, respectively; symmetry codes: (i) *x* − 1, *y*, *z*; (ii) *x* + 1, *y*, *z*].

**Figure 5:**
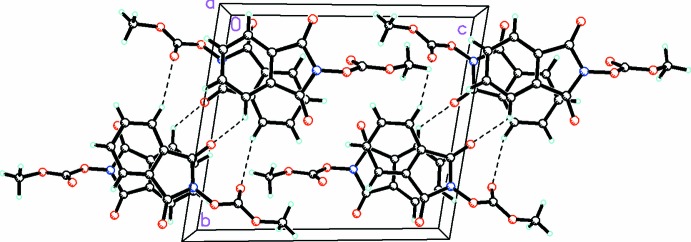
A view along the *a* axis of the crystal packing of compound (2), showing the three-dimensional array formed by an extensive network of C—H⋯O hydrogen bonds (dashed lines; see Table 2 ▶ for details).

In the crystal of (2), the *A* and *B* mol­ecules are linked by C—H⋯O hydrogen bonds (Fig. 5 ▶ and Table 2 ▶), forming slabs parallel to (10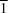). The slabs are in turn linked *via* π–π inter­actions, forming a three-dimensional structure with centroid–centroid distances of 3.4202 (7) for *Cg*1⋯*Cg*5^ii^ and 3.5445 (7) Å for *Cg*2⋯*Cg*4^ii^ [*Cg*1, *Cg*2, *Cg*4 and *C*g5 are the centroids of rings N1*A*/C1*A*/C2*A*/C7*A*/C8*A*, C2*A*–C7*A*, N1*B*/C1*B*/C2*B*/C7*B*/C8*B* and C2*B*–C7*B*, respectively; symmetry code: (ii) *x* + 1, *y*, *z* − 1].

**Figure 6:**
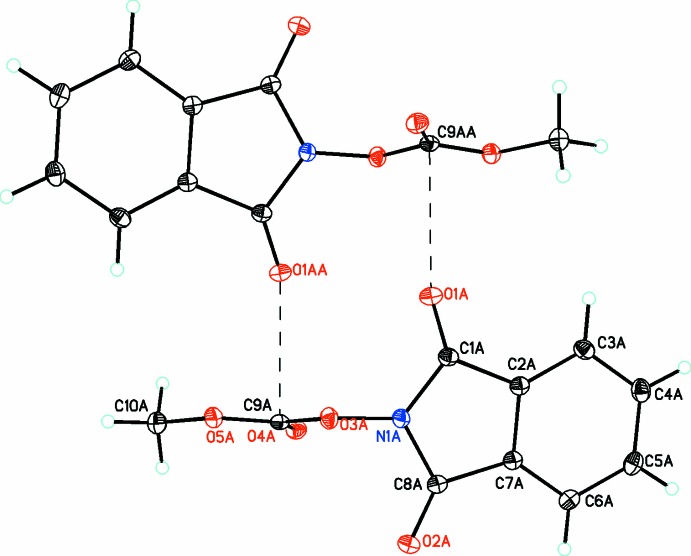
For mol­ecule *A* in compound (2), perpendicular inter­actions between atoms O1*A* and C9*A* (shown as dashed lines) link the mol­ecules into inversion dimers [symmetry code: (A) − *x* + 1, − *y* + 2, −*z*].

In the crystal of (3), the *A* and *B* mol­ecules are linked by C—H⋯O hydrogen bonds (Fig. 6 ▶ and Table 3 ▶), forming a three-dimensional structure, which is consolidated by π–π inter­actions [*Cg*1⋯*Cg*3^iii^ = 3.578 (3), *Cg*2⋯*Cg*3^iii^ = 3.575 (3) Å and *Cg*9⋯*Cg*10^iv^; *Cg*1, *Cg*2, *Cg*3, *Cg*9 and *Cg*10 are the centroids of rings N1*A*/C1*A*–C5*A*, C2*A*/C3*A*/C6*A*–C9*A*, C3*A*/C4*A*/C9*A*–C12*A*, C2*B*/C3*B*/C6*B*–C9*B* and C3*B*/C4*B*/C9*B*–C12*B*, respectively; symmetry codes: (iii) *x*, −*y* + 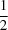, *z* − 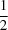; (iv) *x*, −*y* + 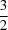, *z* + 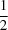].

**Figure 8:**
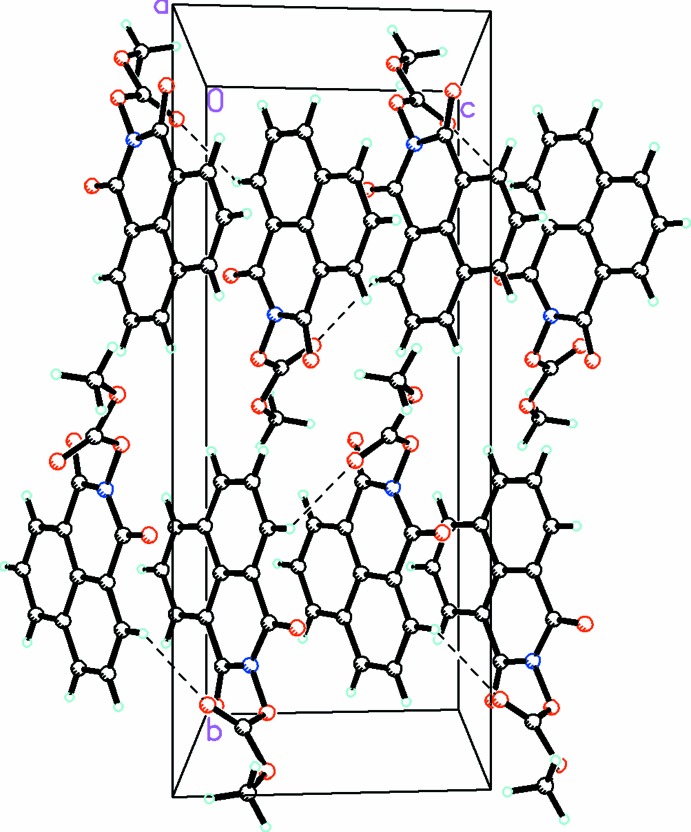
A view along the *a* axis of the crystal packing of compound (3), showing the formation of the three-dimensional array by an extensive network of C—H⋯O hydrogen bonds (dashed lines; see Table 3 ▶ for details).

Inter­estingly, in the crystal of (2) one of the two dione moieties for each mol­ecule (O1*A* and O1*B*) has a short inter­molecular inter­actions with the central C atom of the carbonato group [O1*A*⋯C9*A* = 2.794 (1), O1*B*⋯C9*B* = 2.873 (1) Å], which is perpendicular to the carbonato plane indicating that both atoms, C9*A* and C9*B*, must have significant positive character. These inter­actions link the mol­ecules into dimers as shown in Figs. 6 ▶ and 7 ▶, respectively. This is also noticed to a lesser extent in (3) (Fig. 8 ▶) for mol­ecule *A* (but not for mol­ecule *B*), where a longer inter­molecular inter­action of 3.060 (3) Å is observed between atoms O2*A* and C13*A*, resulting in weakly associated dimers similar to that seen in the case of (2).

## Database survey   

A search of the Cambridge Structural Database (Version 5.35; Groom & Allen, 2014 ▶) for the indoline skeleton gave 26 hits. In all cases, the geometrical parameters of the indoline skeleton are similar to those observed in compounds (1) and (2). In the case of the iso­quinoline structure, there are only two structures containing the planar iso­quinoline moiety with similar geometrical parameters to the present structure, (3).

## Synthesis and crystallization   

**Compound (1):**

To a freshly prepared solution of sodium (2.3 g, 0.10 mol) in absolute ethanol (60 ml) was added a solution of *N*-hy­droxy­phthalimide (16.3 g, 0.10 mol) in absolute ethanol (350 ml), and the red reaction mixture was stirred at room temperature for 30 min. The brick-red precipitate was collected, washed with water, and dried in the oven at 373 K for 30 min to give 17.45 g (95%) of sodium phthalimide oxide as brick-red crystals; m.p. > 573 K. To the solution of sodium phthalimide oxide (0.92 g, 5 mmol) in water (15 ml) was added acetone (10 ml), followed by a solution of bromo­methane (0.66 g, 7 mmol). The reaction mixture was stirred at room temperature for 16 h, during which the red color disappeared. On standing at room temperature for 48 h, the product solidified in the aqueous mixture and was collected. Recrystallization from 2-propanol gave 0.72 g (78%) of compound (1) as plate-like colorless crystals: m.p. 395–397 K; ^1^H NMR (CDC1_3_) δ 3.36 (*s*, 3H, *J* = 6 Hz, OCH_3_), 5.52, *s*, 1 H,CH, 7.87 (*m*, 4 H, phthalimido ring).

**Compound (2):**

To a solution of sodium phthalimide oxide (0.92 g, 5 mmol) in water (15 ml) was added acetone (10 ml), followed by a solution of bromo­(meth­oxy)methanone (0.97 g, 7 mmol). The reaction mixture was stirred at room temperature for 16 h, during which the red color disappeared. On standing at room temperature for 48 h, the product solidified in the aqueous mixture and was collected. Recrystallization from ethanol gave 0.82 g (74%) of compound (2) as colorless crystals: m.p. 410–411 K; ^1^H NMR (CDC1_3_) δ 3.8 (*s*, 3H,OCH_3_), 7.86 (*m*, 4H, phthalimido ring).

**Compound (3):**

To a solution of sodium naphthalimide oxide, (1.18 g, 5 mmol), in water (50 ml), was added bromo­(meth­oxy)methanone (1.25g, 7 mmol) in acetone (10 ml). The red reaction mixture was stirred at room temperature. The red color disappeared within 5 min and the reaction mixture was filled with a white precipitate. After standing for 4 h, the white precipitate was collected, washed with water, and recrystallized from ethanol to give 1.46 g (89%) of compound (3) as colorless crystals: m.p. 483–485 K; ^1^H NMR (CDCl_3_) δ 3.79 (*s*, 3H, OCH_3_), 5.66 (*s*, 1H, CH), 7.65–8.50 (*m*, 6 H, naphthal­imido ring).

## Refinement   

Crystal data, data collection and structure refinement details for (1), (2) and (3) are summarized in Table 4 ▶. For all three compounds, the H atoms were positioned geometrically and refined as riding: C—H = 0.93–0.99 Å with *U_iso_*(H) = 1.5*U*_eq_(C) for methyl H atoms and = 1.2*U_eq_*(C) for other H atoms.

## Supplementary Material

Crystal structure: contains datablock(s) 1, 2, 3. DOI: 10.1107/S1600536814023769/su2795sup1.cif

Structure factors: contains datablock(s) 1. DOI: 10.1107/S1600536814023769/su27951sup2.hkl

Structure factors: contains datablock(s) 2. DOI: 10.1107/S1600536814023769/su27952sup3.hkl

Structure factors: contains datablock(s) 3. DOI: 10.1107/S1600536814023769/su27953sup4.hkl

Supporting information file. DOI: 10.1107/S1600536814023769/su27951sup5.cml

Supporting information file. DOI: 10.1107/S1600536814023769/su27952sup6.cml

Supporting information file. DOI: 10.1107/S1600536814023769/su27953sup7.cml

CCDC references: 1031391, 1031392, 1031393

Additional supporting information:  crystallographic information; 3D view; checkCIF report

## Figures and Tables

**Figure 7:**
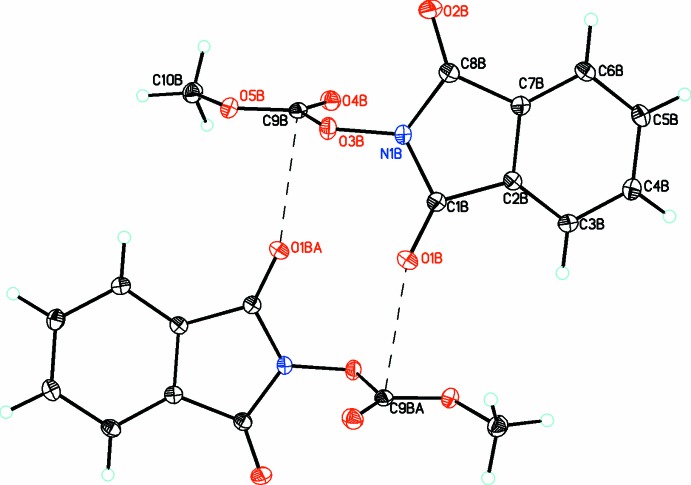
For mol­ecule *B* in compound (2), perpendicular inter­actions between atoms O1*B* and C9*B* (shown as dashed lines) link the mol­ecules into inversion dimers [symmetry code: (A) −*x*, −*y* + 1, −*z* − 1].

**Table 1:** Hydrogen-bond geometry (, ) for (1)

*D*H*A*	*D*H	H*A*	*D* *A*	*D*H*A*
C4H4*A*O2^i^	0.95	2.38	3.190(4)	143
C9H9*A*O1^ii^	0.98	2.54	3.428(7)	151
C9H9*B*O1^iii^	0.98	2.53	3.260(8)	131

Symmetry codes: (i) 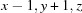; (ii) 
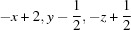
; (iii) 
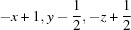
.

**Table 2:** Hydrogen-bond geometry (, ) for (2)

*D*H*A*	*D*H	H*A*	*D* *A*	*D*H*A*
C5*A*H5*AA*O3*B* ^i^	0.95	2.54	3.3341(15)	141
C6*A*H6*AA*O4*A* ^ii^	0.95	2.51	3.4091(15)	158
C3*B*H3*BA*O2*A* ^iii^	0.95	2.59	3.2281(14)	125
C6*B*H6*BA*O3*A* ^iv^	0.95	2.55	3.3086(14)	137
C10*B*H10*F*O2*B* ^v^	0.98	2.57	3.4956(16)	157

Symmetry codes: (i) 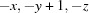; (ii) 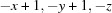; (iii) 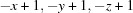; (iv) 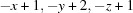; (v) 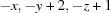.

**Table 3:** Hydrogen-bond geometry (, ) for (3)

*D*H*A*	*D*H	H*A*	*D* *A*	*D*H*A*
C6*A*H6*AA*O4*A* ^i^	0.95	2.51	3.159(5)	125
C7*B*H7*BA*O2*B* ^ii^	0.95	2.51	3.229(5)	133
C10*B*H10*B*O5*B* ^ii^	0.95	2.60	3.428(5)	146
C11*B*H11*B*O1*A* ^iii^	0.95	2.48	3.270(6)	141
C14*A*H14*A*O1*B* ^iv^	0.98	2.51	3.481(5)	169
C14*B*H14*E*O4*A* ^iv^	0.98	2.51	3.306(6)	138

Symmetry codes: (i) 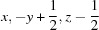; (ii) 
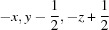
; (iii) 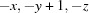; (iv) 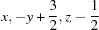.

**Table 4:** Experimental details

	(1)	(2)	(3)
Crystal data			
Chemical formula	C_9_H_7_NO_3_	C_10_H_7_NO_5_	C_14_H_9_NO_5_
*M*_r_	177.16	221.17	271.22
Crystal system, space group	Orthorhombic, *P*2_1_2_1_2_1_	Triclinic, *P* 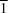	Monoclinic, *P*2_1_/*c*
Temperature (K)	123	123	123
*a*, *b*, *c* ()	4.2987(4), 7.0243(10), 27.587(4)	7.0363(4), 11.0082(5), 12.4239(6)	16.512(3), 18.579(3), 7.6156(13)
, , ()	90, 90, 90	98.884(4), 96.159(4), 93.009(4)	90, 99.434(17), 90
*V* (^3^)	832.98(19)	942.95(8)	2304.6(7)
*Z*	4	4	8
Radiation type	Mo *K*	Cu *K*	Mo *K*
(mm^1^)	0.11	1.10	0.12
Crystal size (mm)	0.66 0.23 0.04	0.35 0.25 0.08	0.44 0.12 0.07

Data collection			
Diffractometer	Agilent Xcalibur (Ruby, Gemini)	SuperNova (Dual, Cu at zero, Atlas)	Agilent Xcalibur (Ruby, Gemini)
Absorption correction	Analytical (*CrysAlis PRO*; Agilent, 2012 ▶)	Multi-scan (*CrysAlis PRO*; Agilent, 2012 ▶)	Analytical (*CrysAlis PRO*; Agilent, 2012 ▶)
*T*_min_, *T*_max_	0.946, 0.996	0.807, 1.000	0.995, 0.999
No. of measured, independent and observed [*I* > 2(*I*)] reflections	5145, 2259, 1989	6437, 3803, 3516	9949, 4156, 1898
*R*_int_	0.087	0.018	0.091
(sin /)_max_ (^1^)	0.727	0.631	0.600

Refinement			
*R*[*F* ^2^ > 2(*F* ^2^)], *wR*(*F* ^2^), *S*	0.099, 0.229, 1.13	0.033, 0.089, 1.06	0.080, 0.224, 1.00
No. of reflections	2259	3803	4156
No. of parameters	119	291	363
H-atom treatment	H-atom parameters constrained	H-atom parameters constrained	H-atom parameters constrained
_max_, _min_ (e ^3^)	0.50, 0.34	0.29, 0.21	0.33, 0.39

Computer programs: *CrysAlis PRO* (Agilent, 2012 ▶), *SUPERFLIP* (Palatinus Chapuis, 2007 ▶), *SHELXS2013*, *SHELXL2013* and *SHELXTL* (Sheldrick, 2008 ▶) and *SUPERFLIP* (Palatinus *et al.* 2007 ▶).

## supplementary crystallographic information

### Crystal data

**Table:**

C_14_H_9_NO_5_	*F*(000) = 1120
*M**_r_* = 271.22	*D*_x_ = 1.563 Mg m^−^^3^
Monoclinic, *P*2_1_/*c*	Mo *K*α radiation, λ = 0.71073 Å
*a* = 16.512 (3) Å	Cell parameters from 1261 reflections
*b* = 18.579 (3) Å	θ = 3.4–26.9°
*c* = 7.6156 (13) Å	µ = 0.12 mm^−^^1^
β = 99.434 (17)°	*T* = 123 K
*V* = 2304.6 (7) Å^3^	Needle, colorless
*Z* = 8	0.44 × 0.12 × 0.07 mm

### Data collection

**Table:**

Agilent Xcalibur (Ruby, Gemini) diffractometer	4156 independent reflections
Radiation source: Enhance (Mo) X-ray Source	1898 reflections with *I* > 2σ(*I*)
Detector resolution: 10.5081 pixels mm^-1^	*R*_int_ = 0.091
ω scans	θ_max_ = 25.3°, θ_min_ = 3.3°
Absorption correction: analytical (*CrysAlis PRO*; Agilent, 2012)	*h* = −15→19
*T*_min_ = 0.995, *T*_max_ = 0.999	*k* = −22→21
9949 measured reflections	*l* = −9→9

### Refinement

**Table:**

Refinement on *F*^2^	0 restraints
Least-squares matrix: full	Hydrogen site location: inferred from neighbouring sites
*R*[*F*^2^ > 2σ(*F*^2^)] = 0.080	H-atom parameters constrained
*wR*(*F*^2^) = 0.224	*w* = 1/[σ^2^(*F*_o_^2^) + (0.0796*P*)^2^] where *P* = (*F*_o_^2^ + 2*F*_c_^2^)/3
*S* = 1.00	(Δ/σ)_max_ < 0.001
4156 reflections	Δρ_max_ = 0.33 e Å^−^^3^
363 parameters	Δρ_min_ = −0.39 e Å^−^^3^

### Special details

**Table:**

Geometry. All e.s.d.'s (except the e.s.d. in the dihedral angle between two l.s. planes) are estimated using the full covariance matrix. The cell e.s.d.'s are taken into account individually in the estimation of e.s.d.'s in distances, angles and torsion angles; correlations between e.s.d.'s in cell parameters are only used when they are defined by crystal symmetry. An approximate (isotropic) treatment of cell e.s.d.'s is used for estimating e.s.d.'s involving l.s. planes.

### Fractional atomic coordinates and isotropic or equivalent isotropic displacement parameters (Å^2^)

**Table:**

	*x*	*y*	*z*	*U*_iso_*/*U*_eq_	
O1A	0.3491 (2)	0.31532 (15)	0.1257 (5)	0.0428 (9)	
O2A	0.5630 (2)	0.44236 (15)	0.4206 (4)	0.0441 (9)	
O3A	0.4152 (2)	0.43894 (14)	0.2299 (4)	0.0384 (9)	
O4A	0.3270 (2)	0.41710 (15)	0.4258 (4)	0.0413 (9)	
O5A	0.31114 (19)	0.50794 (14)	0.2257 (4)	0.0367 (8)	
N1A	0.4539 (3)	0.37502 (17)	0.2925 (5)	0.0375 (10)	
C1A	0.4144 (3)	0.3116 (2)	0.2245 (7)	0.0370 (12)	
C2A	0.4578 (3)	0.2448 (2)	0.2868 (6)	0.0334 (11)	
C3A	0.5339 (3)	0.2484 (2)	0.4016 (6)	0.0335 (11)	
C4A	0.5716 (3)	0.3145 (2)	0.4574 (6)	0.0359 (12)	
C5A	0.5329 (3)	0.3829 (2)	0.3934 (6)	0.0344 (12)	
C6A	0.4237 (3)	0.1799 (2)	0.2309 (6)	0.0361 (12)	
H6AA	0.3722	0.1785	0.1543	0.043*	
C7A	0.4646 (3)	0.1149 (2)	0.2864 (7)	0.0400 (13)	
H7AA	0.4415	0.0700	0.2451	0.048*	
C8A	0.5375 (3)	0.1174 (2)	0.3996 (7)	0.0369 (12)	
H8AA	0.5642	0.0735	0.4376	0.044*	
C9A	0.5748 (3)	0.1828 (2)	0.4622 (7)	0.0365 (12)	
C10A	0.6507 (3)	0.1873 (2)	0.5762 (7)	0.0411 (13)	
H10A	0.6786	0.1443	0.6171	0.049*	
C11A	0.6852 (3)	0.2519 (2)	0.6293 (6)	0.0385 (12)	
H11A	0.7365	0.2534	0.7069	0.046*	
C12A	0.6457 (3)	0.3158 (2)	0.5705 (6)	0.0378 (12)	
H12A	0.6701	0.3606	0.6087	0.045*	
C13A	0.3473 (3)	0.4514 (2)	0.3079 (7)	0.0354 (12)	
C14A	0.2353 (3)	0.5276 (2)	0.2859 (6)	0.0427 (13)	
H14A	0.2134	0.5717	0.2253	0.064*	
H14B	0.2459	0.5358	0.4147	0.064*	
H14C	0.1953	0.4886	0.2583	0.064*	
O1B	0.1669 (2)	0.82517 (15)	0.5198 (4)	0.0392 (8)	
O2B	−0.0410 (2)	0.95254 (15)	0.2005 (4)	0.0468 (10)	
O3B	0.1021 (2)	0.94818 (14)	0.4052 (4)	0.0384 (9)	
O4B	0.1866 (2)	0.92546 (16)	0.2003 (4)	0.0434 (9)	
O5B	0.1998 (2)	1.02184 (14)	0.3847 (4)	0.0392 (9)	
N1B	0.0622 (3)	0.88420 (17)	0.3479 (5)	0.0363 (10)	
C1B	0.1017 (3)	0.8213 (2)	0.4202 (7)	0.0359 (12)	
C2B	0.0594 (3)	0.7545 (2)	0.3564 (6)	0.0325 (11)	
C3B	−0.0186 (3)	0.7576 (2)	0.2461 (6)	0.0358 (12)	
C4B	−0.0567 (3)	0.8244 (2)	0.1926 (6)	0.0349 (12)	
C5B	−0.0156 (3)	0.8933 (2)	0.2436 (7)	0.0376 (12)	
C6B	0.0945 (3)	0.6896 (2)	0.4060 (6)	0.0363 (12)	
H6BA	0.1462	0.6879	0.4820	0.044*	
C7B	0.0547 (3)	0.6251 (2)	0.3450 (7)	0.0392 (13)	
H7BA	0.0803	0.5802	0.3780	0.047*	
C8B	−0.0194 (3)	0.6266 (2)	0.2403 (7)	0.0400 (13)	
H8BA	−0.0454	0.5825	0.2016	0.048*	
C9B	−0.0595 (3)	0.6923 (2)	0.1864 (6)	0.0356 (12)	
C10B	−0.1376 (3)	0.6966 (2)	0.0811 (7)	0.0432 (13)	
H10B	−0.1652	0.6535	0.0395	0.052*	
C11B	−0.1745 (3)	0.7611 (2)	0.0371 (7)	0.0459 (13)	
H11B	−0.2282	0.7626	−0.0307	0.055*	
C12B	−0.1335 (3)	0.8257 (2)	0.0917 (7)	0.0415 (13)	
H12B	−0.1592	0.8705	0.0585	0.050*	
C13B	0.1661 (3)	0.9614 (2)	0.3143 (7)	0.0365 (12)	
C14B	0.2722 (3)	1.0450 (2)	0.3140 (7)	0.0454 (14)	
H14D	0.2953	1.0880	0.3779	0.068*	
H14E	0.2569	1.0562	0.1871	0.068*	
H14F	0.3132	1.0063	0.3292	0.068*	

### Atomic displacement parameters (Å^2^)

**Table:**

	*U*^11^	*U*^22^	*U*^33^	*U*^12^	*U*^13^	*U*^23^
O1A	0.037 (2)	0.0392 (18)	0.047 (2)	0.0021 (16)	−0.0087 (18)	−0.0011 (15)
O2A	0.044 (2)	0.0267 (16)	0.058 (2)	−0.0019 (16)	−0.0013 (18)	−0.0040 (15)
O3A	0.037 (2)	0.0299 (16)	0.046 (2)	0.0043 (15)	−0.0004 (17)	0.0045 (14)
O4A	0.048 (2)	0.0313 (16)	0.044 (2)	0.0042 (16)	0.0033 (18)	0.0056 (16)
O5A	0.039 (2)	0.0245 (15)	0.045 (2)	0.0049 (15)	0.0022 (17)	0.0062 (14)
N1A	0.038 (3)	0.0219 (19)	0.049 (3)	0.0043 (18)	−0.003 (2)	−0.0012 (17)
C1A	0.041 (4)	0.029 (2)	0.040 (3)	0.001 (2)	0.003 (3)	0.000 (2)
C2A	0.034 (3)	0.026 (2)	0.040 (3)	−0.003 (2)	0.005 (2)	0.001 (2)
C3A	0.036 (3)	0.027 (2)	0.037 (3)	0.002 (2)	0.005 (2)	0.003 (2)
C4A	0.036 (3)	0.032 (2)	0.038 (3)	−0.002 (2)	0.000 (2)	−0.002 (2)
C5A	0.038 (3)	0.031 (3)	0.033 (3)	−0.003 (2)	0.004 (2)	0.000 (2)
C6A	0.036 (3)	0.035 (3)	0.037 (3)	−0.006 (2)	0.005 (2)	−0.001 (2)
C7A	0.043 (4)	0.029 (2)	0.048 (3)	−0.002 (2)	0.010 (3)	−0.006 (2)
C8A	0.043 (4)	0.029 (2)	0.038 (3)	0.004 (2)	0.006 (3)	0.002 (2)
C9A	0.037 (3)	0.029 (2)	0.044 (3)	0.002 (2)	0.007 (3)	−0.005 (2)
C10A	0.039 (3)	0.035 (3)	0.048 (4)	0.007 (2)	0.006 (3)	0.002 (2)
C11A	0.029 (3)	0.045 (3)	0.039 (3)	0.002 (2)	−0.005 (2)	−0.001 (2)
C12A	0.042 (3)	0.034 (2)	0.037 (3)	0.000 (2)	0.004 (3)	−0.009 (2)
C13A	0.036 (3)	0.031 (2)	0.038 (3)	−0.004 (2)	0.003 (3)	−0.003 (2)
C14A	0.041 (3)	0.037 (3)	0.046 (3)	0.002 (2)	−0.002 (3)	0.002 (2)
O1B	0.035 (2)	0.0361 (17)	0.044 (2)	0.0022 (16)	−0.0005 (17)	−0.0033 (15)
O2B	0.050 (3)	0.0270 (17)	0.059 (3)	0.0022 (16)	−0.0037 (19)	0.0046 (15)
O3B	0.040 (2)	0.0257 (16)	0.048 (2)	−0.0046 (15)	0.0019 (18)	−0.0046 (14)
O4B	0.050 (2)	0.0384 (18)	0.041 (2)	−0.0032 (16)	0.0047 (18)	−0.0054 (16)
O5B	0.046 (2)	0.0265 (16)	0.043 (2)	−0.0059 (15)	0.0031 (17)	−0.0044 (14)
N1B	0.037 (3)	0.0213 (19)	0.046 (3)	−0.0027 (18)	−0.006 (2)	−0.0020 (17)
C1B	0.033 (3)	0.036 (3)	0.037 (3)	0.005 (2)	0.002 (3)	0.001 (2)
C2B	0.035 (3)	0.027 (2)	0.035 (3)	0.001 (2)	0.002 (2)	−0.007 (2)
C3B	0.040 (3)	0.030 (2)	0.038 (3)	0.001 (2)	0.005 (2)	0.003 (2)
C4B	0.033 (3)	0.032 (2)	0.038 (3)	−0.004 (2)	0.001 (2)	0.000 (2)
C5B	0.040 (4)	0.032 (3)	0.041 (3)	−0.001 (2)	0.006 (3)	−0.002 (2)
C6B	0.039 (3)	0.031 (2)	0.039 (3)	0.001 (2)	0.008 (2)	0.003 (2)
C7B	0.047 (4)	0.025 (2)	0.046 (3)	0.002 (2)	0.010 (3)	0.003 (2)
C8B	0.040 (4)	0.030 (2)	0.050 (4)	−0.004 (2)	0.006 (3)	−0.002 (2)
C9B	0.037 (3)	0.036 (3)	0.032 (3)	−0.005 (2)	0.003 (2)	−0.002 (2)
C10B	0.041 (4)	0.037 (3)	0.049 (3)	−0.009 (2)	0.000 (3)	0.005 (2)
C11B	0.042 (3)	0.048 (3)	0.045 (3)	−0.006 (3)	−0.001 (3)	0.001 (2)
C12B	0.040 (4)	0.037 (3)	0.046 (3)	0.001 (2)	0.002 (3)	0.006 (2)
C13B	0.036 (3)	0.030 (3)	0.042 (3)	0.002 (2)	0.000 (3)	0.007 (2)
C14B	0.044 (4)	0.037 (3)	0.055 (4)	−0.010 (2)	0.007 (3)	0.001 (2)

### Geometric parameters (Å, º)

**Table:**

O1A—C1A	1.211 (6)	O1B—C1B	1.212 (5)
O2A—C5A	1.215 (5)	O2B—C5B	1.204 (5)
O3A—C13A	1.371 (5)	O3B—C13B	1.377 (5)
O3A—N1A	1.395 (4)	O3B—N1B	1.394 (4)
O4A—C13A	1.193 (5)	O4B—C13B	1.188 (5)
O5A—C13A	1.316 (5)	O5B—C13B	1.326 (5)
O5A—C14A	1.449 (5)	O5B—C14B	1.455 (5)
N1A—C1A	1.403 (6)	N1B—C5B	1.405 (6)
N1A—C5A	1.408 (6)	N1B—C1B	1.406 (6)
C1A—C2A	1.473 (6)	C1B—C2B	1.468 (6)
C2A—C6A	1.370 (6)	C2B—C6B	1.365 (6)
C2A—C3A	1.409 (6)	C2B—C3B	1.419 (6)
C3A—C4A	1.410 (6)	C3B—C4B	1.419 (6)
C3A—C9A	1.432 (6)	C3B—C9B	1.427 (6)
C4A—C12A	1.377 (7)	C4B—C12B	1.371 (7)
C4A—C5A	1.471 (6)	C4B—C5B	1.472 (6)
C6A—C7A	1.413 (6)	C6B—C7B	1.408 (6)
C6A—H6AA	0.9500	C6B—H6BA	0.9500
C7A—C8A	1.362 (7)	C7B—C8B	1.347 (7)
C7A—H7AA	0.9500	C7B—H7BA	0.9500
C8A—C9A	1.410 (6)	C8B—C9B	1.417 (6)
C8A—H8AA	0.9500	C8B—H8BA	0.9500
C9A—C10A	1.405 (7)	C9B—C10B	1.404 (7)
C10A—C11A	1.361 (6)	C10B—C11B	1.362 (6)
C10A—H10A	0.9500	C10B—H10B	0.9500
C11A—C12A	1.392 (6)	C11B—C12B	1.406 (6)
C11A—H11A	0.9500	C11B—H11B	0.9500
C12A—H12A	0.9500	C12B—H12B	0.9500
C14A—H14A	0.9800	C14B—H14D	0.9800
C14A—H14B	0.9800	C14B—H14E	0.9800
C14A—H14C	0.9800	C14B—H14F	0.9800
			
C13A—O3A—N1A	110.9 (3)	C13B—O3B—N1B	111.0 (3)
C13A—O5A—C14A	113.6 (3)	C13B—O5B—C14B	114.6 (4)
O3A—N1A—C1A	115.4 (4)	O3B—N1B—C5B	114.5 (3)
O3A—N1A—C5A	115.4 (3)	O3B—N1B—C1B	114.9 (4)
C1A—N1A—C5A	128.4 (4)	C5B—N1B—C1B	130.2 (4)
O1A—C1A—N1A	119.6 (4)	O1B—C1B—N1B	120.2 (4)
O1A—C1A—C2A	125.8 (4)	O1B—C1B—C2B	125.7 (4)
N1A—C1A—C2A	114.6 (5)	N1B—C1B—C2B	114.0 (5)
C6A—C2A—C3A	120.9 (4)	C6B—C2B—C3B	120.2 (4)
C6A—C2A—C1A	119.3 (5)	C6B—C2B—C1B	119.8 (5)
C3A—C2A—C1A	119.8 (4)	C3B—C2B—C1B	119.9 (4)
C2A—C3A—C4A	122.2 (4)	C2B—C3B—C4B	121.4 (4)
C2A—C3A—C9A	119.0 (4)	C2B—C3B—C9B	119.3 (4)
C4A—C3A—C9A	118.8 (5)	C4B—C3B—C9B	119.2 (5)
C12A—C4A—C3A	120.5 (4)	C12B—C4B—C3B	120.1 (4)
C12A—C4A—C5A	119.1 (4)	C12B—C4B—C5B	118.5 (4)
C3A—C4A—C5A	120.4 (5)	C3B—C4B—C5B	121.4 (5)
O2A—C5A—N1A	120.3 (4)	O2B—C5B—N1B	120.7 (4)
O2A—C5A—C4A	125.9 (5)	O2B—C5B—C4B	126.7 (5)
N1A—C5A—C4A	113.8 (4)	N1B—C5B—C4B	112.5 (4)
C2A—C6A—C7A	120.5 (5)	C2B—C6B—C7B	120.4 (5)
C2A—C6A—H6AA	119.7	C2B—C6B—H6BA	119.8
C7A—C6A—H6AA	119.7	C7B—C6B—H6BA	119.8
C8A—C7A—C6A	119.3 (4)	C8B—C7B—C6B	120.5 (4)
C8A—C7A—H7AA	120.3	C8B—C7B—H7BA	119.8
C6A—C7A—H7AA	120.3	C6B—C7B—H7BA	119.8
C7A—C8A—C9A	122.3 (4)	C7B—C8B—C9B	121.7 (4)
C7A—C8A—H8AA	118.8	C7B—C8B—H8BA	119.1
C9A—C8A—H8AA	118.8	C9B—C8B—H8BA	119.1
C10A—C9A—C8A	123.8 (4)	C10B—C9B—C8B	123.8 (4)
C10A—C9A—C3A	118.3 (4)	C10B—C9B—C3B	118.4 (4)
C8A—C9A—C3A	117.9 (5)	C8B—C9B—C3B	117.8 (5)
C11A—C10A—C9A	121.6 (4)	C11B—C10B—C9B	121.5 (5)
C11A—C10A—H10A	119.2	C11B—C10B—H10B	119.2
C9A—C10A—H10A	119.2	C9B—C10B—H10B	119.2
C10A—C11A—C12A	120.3 (5)	C10B—C11B—C12B	120.2 (5)
C10A—C11A—H11A	119.9	C10B—C11B—H11B	119.9
C12A—C11A—H11A	119.9	C12B—C11B—H11B	119.9
C4A—C12A—C11A	120.5 (4)	C4B—C12B—C11B	120.5 (5)
C4A—C12A—H12A	119.7	C4B—C12B—H12B	119.8
C11A—C12A—H12A	119.7	C11B—C12B—H12B	119.8
O4A—C13A—O5A	128.5 (4)	O4B—C13B—O5B	128.3 (5)
O4A—C13A—O3A	126.0 (4)	O4B—C13B—O3B	126.9 (4)
O5A—C13A—O3A	105.6 (4)	O5B—C13B—O3B	104.7 (4)
O5A—C14A—H14A	109.5	O5B—C14B—H14D	109.5
O5A—C14A—H14B	109.5	O5B—C14B—H14E	109.5
H14A—C14A—H14B	109.5	H14D—C14B—H14E	109.5
O5A—C14A—H14C	109.5	O5B—C14B—H14F	109.5
H14A—C14A—H14C	109.5	H14D—C14B—H14F	109.5
H14B—C14A—H14C	109.5	H14E—C14B—H14F	109.5
			
C13A—O3A—N1A—C1A	75.2 (5)	C13B—O3B—N1B—C5B	−107.2 (4)
C13A—O3A—N1A—C5A	−113.9 (4)	C13B—O3B—N1B—C1B	79.7 (5)
O3A—N1A—C1A—O1A	−3.6 (6)	O3B—N1B—C1B—O1B	−2.2 (6)
C5A—N1A—C1A—O1A	−173.0 (5)	C5B—N1B—C1B—O1B	−173.9 (5)
O3A—N1A—C1A—C2A	177.8 (4)	O3B—N1B—C1B—C2B	−179.2 (3)
C5A—N1A—C1A—C2A	8.3 (7)	C5B—N1B—C1B—C2B	9.1 (7)
O1A—C1A—C2A—C6A	0.3 (8)	O1B—C1B—C2B—C6B	−1.7 (8)
N1A—C1A—C2A—C6A	178.8 (4)	N1B—C1B—C2B—C6B	175.1 (4)
O1A—C1A—C2A—C3A	179.8 (5)	O1B—C1B—C2B—C3B	177.2 (4)
N1A—C1A—C2A—C3A	−1.6 (6)	N1B—C1B—C2B—C3B	−6.0 (7)
C6A—C2A—C3A—C4A	178.2 (4)	C6B—C2B—C3B—C4B	180.0 (4)
C1A—C2A—C3A—C4A	−1.3 (7)	C1B—C2B—C3B—C4B	1.1 (7)
C6A—C2A—C3A—C9A	−1.1 (7)	C6B—C2B—C3B—C9B	−0.5 (7)
C1A—C2A—C3A—C9A	179.4 (4)	C1B—C2B—C3B—C9B	−179.4 (4)
C2A—C3A—C4A—C12A	179.6 (4)	C2B—C3B—C4B—C12B	−177.4 (4)
C9A—C3A—C4A—C12A	−1.1 (7)	C9B—C3B—C4B—C12B	3.1 (7)
C2A—C3A—C4A—C5A	−1.3 (7)	C2B—C3B—C4B—C5B	2.3 (7)
C9A—C3A—C4A—C5A	178.0 (4)	C9B—C3B—C4B—C5B	−177.2 (4)
O3A—N1A—C5A—O2A	−0.2 (7)	O3B—N1B—C5B—O2B	4.6 (6)
C1A—N1A—C5A—O2A	169.3 (4)	C1B—N1B—C5B—O2B	176.3 (4)
O3A—N1A—C5A—C4A	179.8 (3)	O3B—N1B—C5B—C4B	−177.6 (3)
C1A—N1A—C5A—C4A	−10.7 (7)	C1B—N1B—C5B—C4B	−5.9 (7)
C12A—C4A—C5A—O2A	5.6 (8)	C12B—C4B—C5B—O2B	−3.0 (8)
C3A—C4A—C5A—O2A	−173.4 (5)	C3B—C4B—C5B—O2B	177.3 (5)
C12A—C4A—C5A—N1A	−174.3 (4)	C12B—C4B—C5B—N1B	179.4 (4)
C3A—C4A—C5A—N1A	6.6 (7)	C3B—C4B—C5B—N1B	−0.3 (7)
C3A—C2A—C6A—C7A	−0.5 (7)	C3B—C2B—C6B—C7B	1.4 (7)
C1A—C2A—C6A—C7A	179.0 (4)	C1B—C2B—C6B—C7B	−179.7 (4)
C2A—C6A—C7A—C8A	1.5 (7)	C2B—C6B—C7B—C8B	−1.5 (7)
C6A—C7A—C8A—C9A	−0.9 (7)	C6B—C7B—C8B—C9B	0.6 (8)
C7A—C8A—C9A—C10A	−178.7 (5)	C7B—C8B—C9B—C10B	−178.4 (5)
C7A—C8A—C9A—C3A	−0.6 (7)	C7B—C8B—C9B—C3B	0.3 (7)
C2A—C3A—C9A—C10A	179.8 (4)	C2B—C3B—C9B—C10B	178.4 (4)
C4A—C3A—C9A—C10A	0.5 (7)	C4B—C3B—C9B—C10B	−2.1 (7)
C2A—C3A—C9A—C8A	1.6 (7)	C2B—C3B—C9B—C8B	−0.4 (7)
C4A—C3A—C9A—C8A	−177.7 (4)	C4B—C3B—C9B—C8B	179.2 (4)
C8A—C9A—C10A—C11A	178.2 (4)	C8B—C9B—C10B—C11B	178.1 (5)
C3A—C9A—C10A—C11A	0.2 (8)	C3B—C9B—C10B—C11B	−0.6 (8)
C9A—C10A—C11A—C12A	−0.3 (7)	C9B—C10B—C11B—C12B	2.3 (8)
C3A—C4A—C12A—C11A	1.0 (8)	C3B—C4B—C12B—C11B	−1.4 (8)
C5A—C4A—C12A—C11A	−178.0 (4)	C5B—C4B—C12B—C11B	178.9 (4)
C10A—C11A—C12A—C4A	−0.3 (7)	C10B—C11B—C12B—C4B	−1.3 (8)
C14A—O5A—C13A—O4A	−2.6 (7)	C14B—O5B—C13B—O4B	−1.5 (7)
C14A—O5A—C13A—O3A	177.5 (3)	C14B—O5B—C13B—O3B	177.3 (3)
N1A—O3A—C13A—O4A	6.8 (6)	N1B—O3B—C13B—O4B	0.7 (7)
N1A—O3A—C13A—O5A	−173.3 (3)	N1B—O3B—C13B—O5B	−178.1 (3)

### Hydrogen-bond geometry (Å, º)

**Table:**

*D*—H···*A*	*D*—H	H···*A*	*D*···*A*	*D*—H···*A*
C6*A*—H6*AA*···O4*A*^i^	0.95	2.51	3.159 (5)	125
C7*B*—H7*BA*···O2*B*^ii^	0.95	2.51	3.229 (5)	133
C10*B*—H10*B*···O5*B*^ii^	0.95	2.60	3.428 (5)	146
C11*B*—H11*B*···O1*A*^iii^	0.95	2.48	3.270 (6)	141
C14*A*—H14*A*···O1*B*^iv^	0.98	2.51	3.481 (5)	169
C14*B*—H14*E*···O4*A*^iv^	0.98	2.51	3.306 (6)	138

Symmetry codes: (i) *x*, −*y*+1/2, *z*−1/2; (ii) −*x*, *y*−1/2, −*z*+1/2; (iii) −*x*, −*y*+1, −*z*; (iv) *x*, −*y*+3/2, *z*−1/2.
